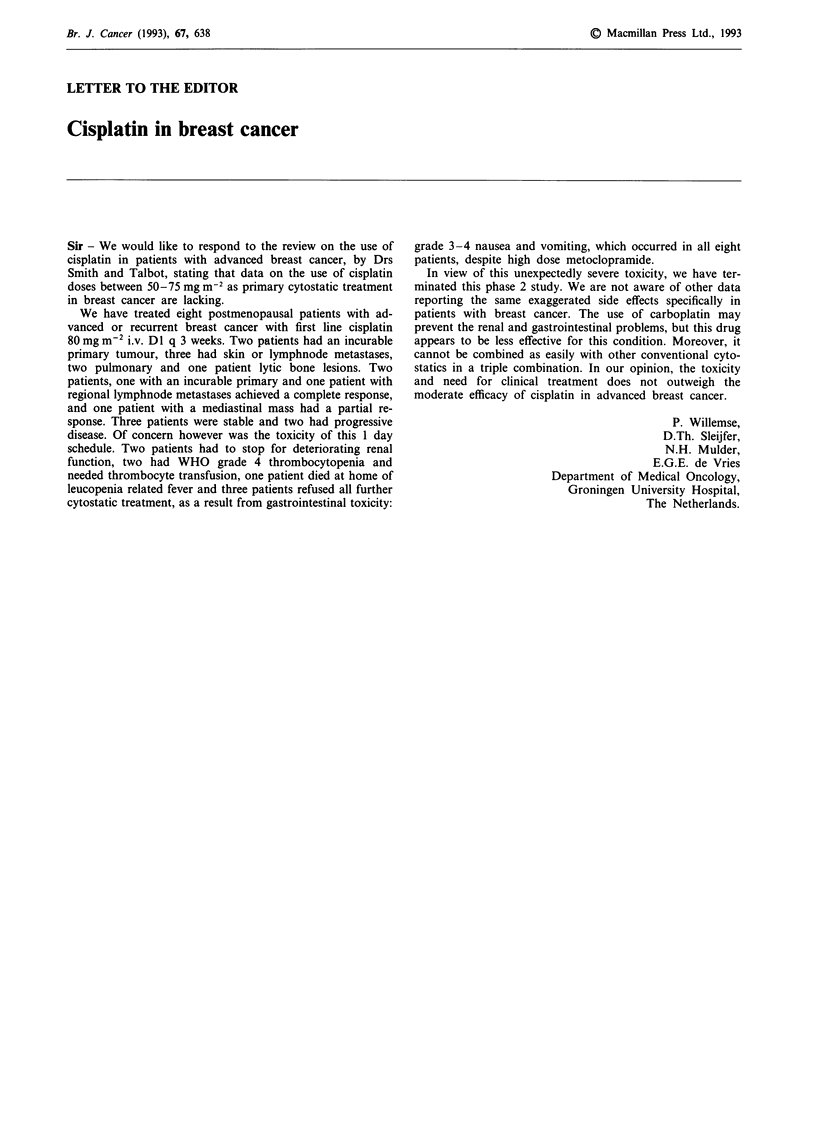# Cisplatin in breast cancer.

**DOI:** 10.1038/bjc.1993.117

**Published:** 1993-03

**Authors:** P. Willemse, D. T. Sleijfer, N. H. Mulder, E. G. de Vries


*Br. J. Cancer* (1993), **67**, 638 © Macmillan Press Ltd., 1993

**LETTER TO THE EDITOR**

### Cisplatin in breast cancer

**Sir** – We would like to respond to the review on the use of cisplatin in patients with advanced breast cancer, by Drs Smith and Talbot, stating that data on the use of cisplatin doses between 50–75 mg $m^{-2}$ as primary cytostatic treatment in breast cancer are lacking.

We have treated eight postmenopausal patients with advanced or recurrent breast cancer with first line cisplatin 80 mg $m^{-2}$ i.v. D1 q 3 weeks. Two patients had an incurable primary tumour, three had skin or lymphnode metastases, two pulmonary and one patient lytic bone lesions. Two patients, one with an incurable primary and one patient with regional lymphnode metastases achieved a complete response, and one patient with a mediastinal mass had a partial response. Three patients were stable and two had progressive disease. Of concern however was the toxicity of this 1 day schedule. Two patients had to stop for deteriorating renal function, two had WHO grade 4 thrombocytopenia and needed thrombocyte transfusion, one patient died at home of leucopenia related fever and three patients refused all further cytostatic treatment, as a result from gastrointestinal toxicity: grade 3–4 nausea and vomiting, which occurred in all eight patients, despite high dose metoclopramide.

In view of this unexpectedly severe toxicity, we have terminated this phase 2 study. We are not aware of other data reporting the same exaggerated side effects specifically in patients with breast cancer. The use of carboplatin may prevent the renal and gastrointestinal problems, but this drug appears to be less effective for this condition. Moreover, it cannot be combined as easily with other conventional cytostatics in a triple combination. In our opinion, the toxicity and need for clinical treatment does not outweigh the moderate efficacy of cisplatin in advanced breast cancer.

P. Willemse,
D.Th. Sleijfer,
N.H. Mulder,
E.G.E. de Vries
Department of Medical Oncology,
Groningen University Hospital,
The Netherlands.